# Optimizing the Intralayer and Interlayer Compatibility for High-Efficiency Blue Thermally Activated Delayed Fluorescence Diodes

**DOI:** 10.1038/srep19904

**Published:** 2016-01-29

**Authors:** Chunbo Duan, Chaochao Fan, Ying Wei, Fuquan Han, Wei Huang, Hui Xu

**Affiliations:** 1 Key Laboratory of Functional Inorganic Material Chemistry, Ministry of Education, Heilongjiang University, 74 Xuefu Road, Harbin 150080, P. R. China; 2 Key Laboratory of Flexible Electronics (KLOFE) & Institute of Advanced Materials (IAM), Jiangsu National Synergetic Innovation Center for Advanced Materials (SICAM), Nanjing Tech University (NanjingTech), 30 South Puzhu Road, Nanjing 211816, P.R. China

## Abstract

A series of phosphine oxide hosts, 4,6-bis(diphenylphosphoryl) dibenzothiophene (**DBTDPO**) and 4- diphenylphosphoryldibenzothiophene (**DBTSPO**), and electron transporting materials (ETM), 2-(diphenylphosphoryl)dibenzothiophene sulfone (**2DBSOSPO**), 3-(diphenylphosphoryl)dibenzothiophene sulfone (**3DBSOSPO**) and 4-(diphenylphosphoryl)dibenzothiophene sulfone (**4DBSOSPO**) were developed to support blue thermally activated delayed fluorescence (TADF) devices with high performance through optimizing intralayer and interlayer compatibility of emissive layers. On the basis of the triplet energy of ~3.0 eV for the hosts and ETMs, excitons can be effectively confined on **DMAC-DPS**. Compared to **DBTSPO**, **DBTDPO** can support the excellent distribution uniformity to blue TADF dye bis[4-(9,9-dimethyl–9,10-dihydroacridine) phenyl] sulfone (**DMAC-DPS**), owing to their configuration similarity; while **3DBSOSPO** and **4DBSOSPO** are superior in compatibility with the hosts due to the similar molecular polarity or configuration. Through adjusting the molecular configuration, the electrical performance of ETMs can be feasibly tuned, including the excellent electron mobility (*μ*_e_) by the order of 10^−3^ cm^2^ V^−1^ s^−1^. As the result, **DBTDPO** and **4DBSOSPO** endowed their four-layer blue TADF devices with the maximum current efficiency of 33.5 cd A^−1^ and the maximum external quantum efficiency more than 17%, which are impressive among the best blue TADF devices. It is showed that intralayer compatibility determines the maximum efficiencies, while interlayer compatibility influences efficiency stability.

Thermally activated delayed fluorescence (TADF) diodes have emerged in recent years as the third-generation organic light-emitting diodes, with the feature of harvesting triplet excitons through pure-organic TADF dyes with efficient reverse intersystem crossing (RISC)^1^,^2^,^3^,^4^. Theoretically, TADF devices almost perfectly combine the respective advantages of fluorescence and phosphorescence organic light-emitting diodes (FLOLEDs and PHOLEDs) in economic and environmental protection and energy conservation, making this kind of light-emitting devices have a profound potential as high-efficiency artificial light sources^5^,^6^,^7^,^8^,^9^,^10^.

Efficient RISC requires singlet-triplet splitting (Δ*E*_ST_) between the first singlet (S_1_) and triplet (T_1_) energy levels as small as possible^11^,^12^,^13^,^14^,^15^,^16^,^17^,^18^,^19^. In this case, the low-energy singlet charge transfer states (^1^CT) are commonly utilized on the basis of strong electronic coupling in donor-acceptor (D-A) systems^18^,^19^,^20^,^21^,^22^,^23^. However, the employment of D-A structures renders the high molecular polarity and strong intermolecular interactions for TADF dyes, remarkably worsening the quenching effects^24^, e.g. triplet-triplet annihilation (TTA)^25^,^26^ and triplet-polaron quenching (TPQ)^27^,^28^. Therefore, most of the efficient TADF devices adopt emitting layers (EML) with doping structures of TADF dyes dispersed in host matrixes^29^,^30^,^31^,^32^,^33^. Similar to PHOLEDs, the utilization of triplet exciton not only endows the 100% theoretical internal quantum efficiency to TADF diodes, but also makes demands on excited-state characteristics of host materials, such as high T_1_ energy for positive energy transfer to dopant and favourable charge injecting/transporting ability for effective charge carrier recombination^34^. In consequence, many conventional phosphorescent host materials, such as N,N′-dicarbazolyl–4,4′-biphenyl (**CBP**)^1^, 1,3-bis(carbazolyl)benzene (***m*****CP**)^35^ and bis[2-[di(phenyl)phosphino]-phenyl]ether oxide (**DPEPO**)^36^, were adopted to realize the efficient red, green, blue and white TADF devices. Nevertheless, since T_1_ value of blue TADF dyes is much higher than their phosphorescent counterparts, e.g. 2.9 eV of bis[4-(9,9-dimethyl–9,10-dihydroacridine)phenyl]sulfone (**DMAC-DPS**)^37^ and 2.75 eV of bis(4,6-(difluorophenyl) pyridinato-*N*,*C*^*2*^)picolinate iridium(III) (**FIrpic**)^38^, it is more challenging to develop high-energy-gap blue TADF host materials with favourable electroactivity. In addition, it is noteworthy that the high molecular polarity of TADF dyes also worsens the interactions with host materials, resulting in the serious host-dopant quenching^39^,^40^,^41^. Ishimatsu and Adachi *et al*. demonstrated the reduction of photoluminescence quantum yield (PLQY) for TADF dyes in polar solvents, due to the increased nonradiative decay of S_1_ state, which reflected the significant influence of the interactions between hosts and TADF dopants on luminescence performance of their solid solutions^42^. In this sense, a promising blue TADF host should possess the high T_1_ energy, good electrical performance and host-dopant compatibility, viz. intralayer compatibility of EML.

On the other hand, the effective exciton confinement in EML for high electroluminescence (EL) efficiencies requires the T_1_ energy of adjacent carrier transporting materials higher than that of dopants, among which the high-energy-gap electron transporting materials (ETM) are all along focused due to the contradiction between strong electron-withdrawing effect and high T_1_ energy^43^,^44^,^45^,^46^,^47^,^48^,^49^,^50^. To achieve the high electron affinity and mobility, ETMs should be established on polar groups, which either reduce excited energy or worsen interfacial interactions^51^. For TADF diodes, the sensitivity of TADF dyes to environmental polarity makes the interfacial interactions between host, dopant and ETM significant for device performance. On account of the charge carrier recombination zones close to EML/electron transporting layer (ETL) interfaces in the conventional hole-predominant devices, exciton can be quenched due to poor interlayer compatibility between EML and ETL, e.g. the formation of interfacial defects, dipole, charge/exciton traps and exciton-polaron interactions^52^,^53^,^54^,^55^,^56^. In our recent work, it was showed that the interfacial interaction-induced quenching effect might be predominant to high efficiencies^57^. Consequently, three prerequisites should be satisfied for high-performance blue TADF ETMs: (i) T_1_ value approaching to 3.0 eV for exciton confinement; (ii) high electron affinity and mobility for charge flux balance and (iii) good compatibility with EML for suppressing interfacial quenching, viz. interlayer compatibility between EML and electron transporting layer (ETL). It is showed that the requirements on the properties of host and ETM are similar but rather difficult to be qualified simultaneously, especially for intralayer and interlayer compatibility of EML. In this case, it is imperative to figure out which one among these requirements is predominant and prioritized for molecular design.

In this contribution, two phosphine oxide hosts, 4,6-bis(diphenylphosphoryl)dibenzothiophene (DBTDPO) and 4- diphenylphosphoryldibenzothiophene (**DBTSPO**), collectively named **DBT*****x*****PO**, and three phosphine oxide ETMs, 2-(diphenylphosphoryl)dibenzothiophene sulfone (**2DBSOSPO**), 3-(diphenylphosphoryl)dibenzothiophene sulfone (**3DBSOSPO**) and 4-(diphenylphosphoryl)dibenzothiophene sulfone (**4DBSOSPO**), collectively named ***m*****DBSOSPO**, were utilized to fabricate blue TADF devices with **DMAC-DPS** as dopant (Fig. 1). Exciton can be effectively confined on the emitter, in virtue of the high T_1_ energy of **DBT*****x*****PO** and ***m*****DBSOSPO** as ~3.0 eV. The frontier molecular orbital (FMO) levels of ***m*****DBSOSPO** are accurately tuned by adjusting substitution position of their diphenylphosphine oxide (DPPO), accompanied with the excellent electron mobility (*μ*_e_) by the order of 10^−3^ cm^2^ V^−1^ s^−1^. Through optimizing the intralayer and interlayer compatibilities of EML, **DBTDPO** and **4DBSOSPO** endowed their four-layer blue TADF devices with the impressive performance of current efficiency (CE) up to 33.5 cd A^−1^ and the maximum external quantum efficiency (EQE) beyond 17%. It is showed that the compatibility between host and dopant has predominant influence on the device efficiency, but the poor interlayer compatibility would worsen efficiency roll-offs due to the serious interfacial quenching effects. This work indicated the significance of intralayer and interlayer compatibility optimization and verified a feasible strategy of high-performance blue TADF diodes.

## Results and Discussions

### Design, Synthesis and Structures

The unique optoelectronic properties of **DBT*****x*****PO** make them superior as the hosts for blue TADF dyes. Their T_1_ values are 2.98 and 2.90 eV to confine triplet excitons on **DMAD-DPS**. Their highest occupied molecular orbital (HOMO) and the lowest unoccupied molecular orbital (LUMO) levels are −6.0 and −2.5 eV. However, even through their dipole moments are also similar as ~4 Debye, symmetrically V-shaped **DBTDPO** is predominant to asymmetric **DBTSPO** in compatibility with **DMAC-DPS**, according to the “similarity-intermiscibility” theory. The surface morphology of the films of **DBT*****x*****PO** before and after **DMAC-DPS** doping can directly manifest host-dopant compatibility. The images of atom force microscopy (AFM) of the neat *vacuum*-evaporated films of **DBT*****x*****PO** (100 nm of thickness) showed the similar surface smoothness with almost equivalent root-mean-square roughness (*R*_s_) of ~0.37 nm, while the film formability of **DMAC-DPS** was poor with *R*_s_ as large as 10 nm, which should be attributed to its strong intermolecular interaction induced aggregation (Fig. 2). After doping, the roughness of **DBTDPO**:**DMAC-DPS** film with concentration of 10%wt. was nearly unchanged, indicating the uniform dispersion of **DMAC-DPS** in **DBTDPO** matrix and their perfect compatibility. Contrarily, *R*_s_ of **DBTSPO**:**DMAC-DPS** film was about two folds of that of neat **DBTSPO** film. The aggregates of **DMAC-DPS** was formed in DBTSPO matrix, reflecting their different molecular symmetry induced poor compatibility.

The electron-donating dibenzothiophene (**DBT**) can be transformed to electron-withdrawing dibenzothiophene sulfone (**DBSO**) after oxidation of S atom. Therefore, the similar structures, different electrical characteristics and homology of **DBT** and **DBSO** can be utilized to construct hosts and ETMs with good compatibility. It is known that when bonding with strong electron-withdrawing groups, phosphine oxide (PO) groups can further polarize the molecules to enhance electrical performance and serve as bridge groups to assist charge transfer. Therefore, through varying DPPO substitution position, the intermolecular interactions between **DBSO** cores of adjacent ***m*****DBSOSPO** molecules can be adjusted in virtue of steric effect of DPPO groups at different substitution positions, giving rise to their different electrical properties. Simultaneously, DPPO substitution positions determine the configurations of ***m*****DBSOSPO** and thereby adjust interlayer compatibility with **DBT*****x*****PO**. According to molecular symmetry, ***m*****DBSOSPO** should be more compatible with **DBTSPO** rather than **DBTDPO**, while **DBTDPO** is superior to **DBTSPO** in the compatibility with **DMAC-DPS**. This discrepancy between **DBT*****x*****PO** in intralayer and interlayer compatibility of EML can reflect which one is predominant to influence device performance.

**Figure 1:**
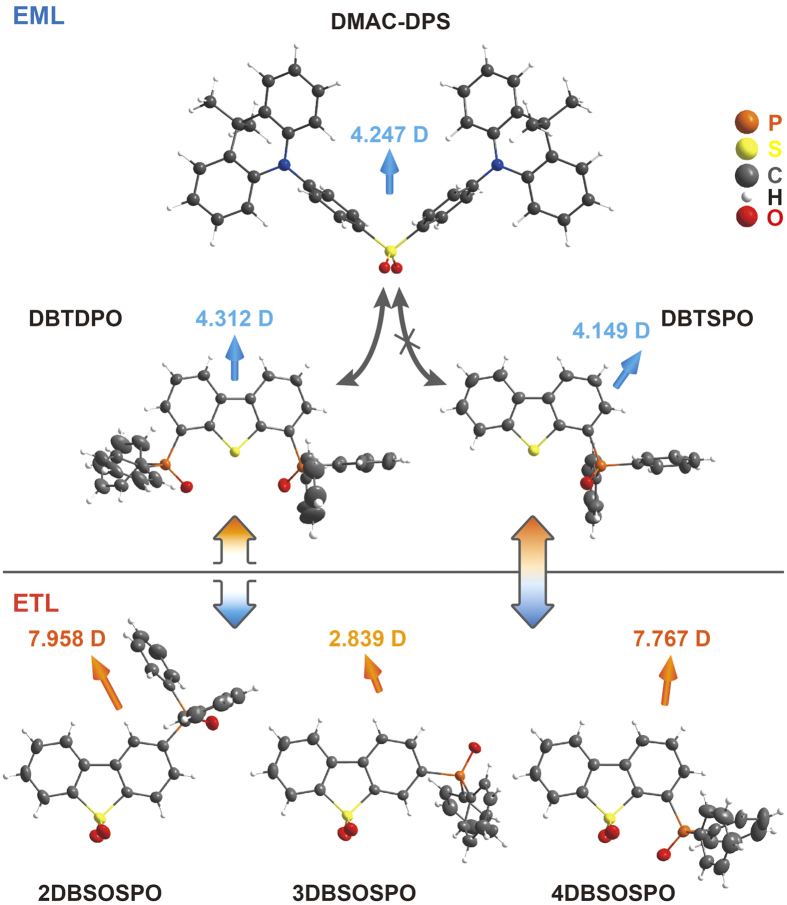
Chemical structures and dipole moments of single crystals of DBT*x*PO and *m*DBSOSPO, as well as those of DMAC-DPS.

***m*****DBSOSPO** can be conveniently synthesized from corresponding bromides of **DBSO** through Pd-catalyzed C-P coupling reaction with moderate total yields of 35–40%, respectively (Fig. 3). The structure characterization was established on the basis of mass spectrometry, NMR spectroscopy and elemental analysis. Their molecular structures are further confirmed by single crystal X-ray diffraction analysis (Fig. 1), in which only ***m*****DBSOSPO** molecules are included. A close inspection reveals the *π*-*π* interactions between **DBSO** cores in adjacent **2DBSOSPO** molecules with a short distance of 3.456 Å, which forms a direct and efficient channel for intermolecular electron hopping (Fig. 4a). The larger steric hindrance of DPPO groups at long axis of **DBSO** core in **3DBSOSPO** enlarges the distance between adjacent **DBSO** cores to 6.659 Å. Nevertheless, two kinds of edge-to-face interactions between two adjacent DPPO groups and **DBSO** core are observed to establish electron transfer channels with the assistance of DPPO as bridge groups (Fig. 4b). Different to **2DBSOSPO** and **3DBSOSPO** with single molecular orientation, two kinds of **4DBSOSPO** molecules exist in its packing diagram (Fig. 4c), in which two parallel **4DBSOSPO** molecules are partitioned by one vertical **4DBSODPO** molecule. There are a series of S = O…H and edge-to-face interactions formed between vertical and parallel **4DBSODPO** molecules, making the former serve as interchange of intermolecular electron hopping. As expected, the different substitution position of DPPO results in the different intermolecular interaction in ***m*****DBSOSPO**, thereby rendering their different electron hopping processes. The **DBSO**-centered channel in **2DBSOSPO** facilitates the direct, consistent and rapid electron transfer. **3DBSOSPO** showed two kinds of electron hopping paths as **DBSO** → DPPO → **DBSO** and **DBSO** → DPPO → DPPO → **DBSO** with one or two DPPOs as bridge groups. The electron migration in **4DBSOSPO** is the most complicated with multiple mutual **DBSO** → DPPO electron hopping. It is rational that the effective electron transportation is in direct proportion to continuity and coherence of intermolecular electron hopping. In this sense, just according to intermolecular interactions, **2DBSOSPO** should be superior in electron transportation among ***m*****DBSOSPO**.

Differential scanning calorimetry (DSC) and thermogravimetric analysis (TGA) of ***m*****DBSOSPO** were performed to investigate their thermal properties (Figure S1 and Table 1). Both the temperature of glass transition (*T*_g_) and melting point (*T*_m_) of **2DBSOSPO** were observed at 151 and 221 °C, indicating its improved morphological stability. *T*_m_ of **3DBSOSPO** decreases to 211 °C, in accord with its weaker intermolecular interactions. However, **4DBSOSPO** revealed the highest *T*_m_ of 264 °C, which should be ascribed to the strong S = O…H interactions. In addition, the decomposition temperatures (*T*_d_) at weight loss of 5% for ***m*****DBSOSPO** were similar as 370 °C, high enough to make device fabrication through *vacuum* evaporation feasible. Different to **2DBSOSPO** and **3DBSOSPO** with single decomposition process, two weight-loss steps were observed for **4DBSOSPO**, indicating its stronger rigidity and intramolecular interactions between **DBSO** and DPPO.

### Optical Properties

**Figure 2:**
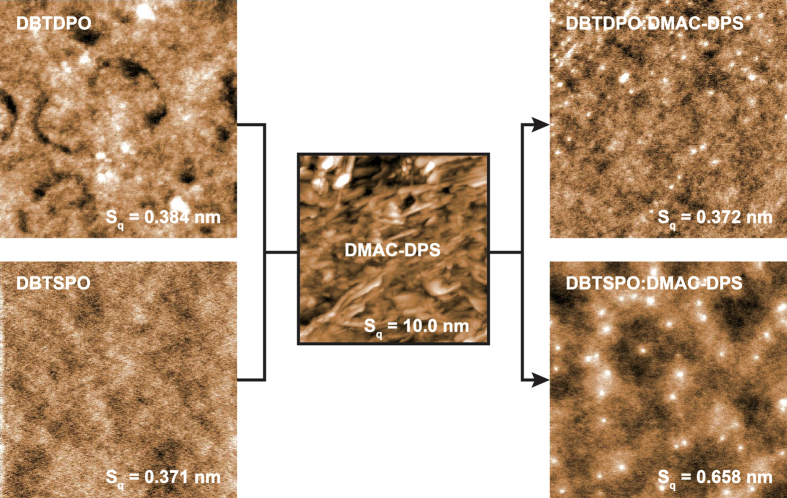
AFM images of vacuum-evaporated thin films of *m*DBSOSPO with thickness of 100 nm before (left) and after (right) DMAC-DPS doping.

**Figure 3:**
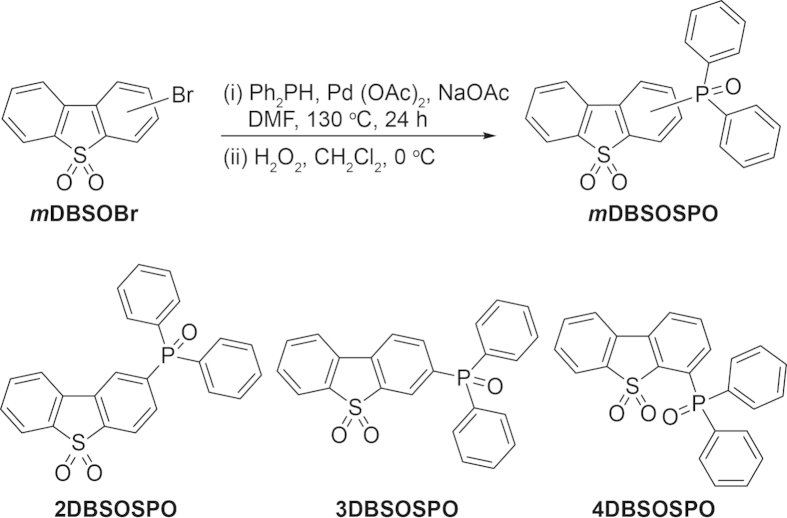
Synthetic procedure of *m*DBSOSPO (*m* = 2, 3 and 4).

The optical properties of **DBT*****x*****PO** are identical with the same fluorescence and phosphorescence spectra. The large-range spectroscopic overlaps between their PL spectra and the absorption spectrum of **DMAC-DPS** from 320 to 400 nm support the efficient energy transfer (Fig. 5a), which is further verified by the identical **DMAC-DPS**-attributed emissions with peaks at 482 nm from *vacuum*-evaporated **DBT*****x*****PO**:**DMAC-DPS** films. However, PLQY of **DBTDPO**:**DMAC-DPS** film reaches to 87%, which is remarkably higher than 75% of **DBTSPO**:**DMAC-DPS** film. The transient PL spectra of these films shows the major contribution of delayed fluorescence component to the sky-blue emissions (inset in Fig. 5a). However, the lifetime of delayed fluorescence (*τ*_DF_) for **DBTSPO**:**DMAC-DPS** is 22.9 *μ*s, about 2 folds of that of **DBTDPO**:**DMAC-DPS** (11.5 *μ*s). Both smaller PLQY and longer *τ*_DF_ of **DBTSPO**:**DMAC-DPS** film should be attributed to the remarkable aggregation of **DMAC-DPS**, as indicated by its AFM image (Fig. 2). The too long emission lifetime would worsen the quenching effect in **DBTSPO**:**DMAC-DPS**, let alone its inferiority in PLQY.

Electronic absorption spectra of ***m*****DBSOSPO** in dilute solutions (10^−6^ mol L^−1^ in CH_2_Cl_2_) are almost identical to that of **DBSO**, except for the peaks at 230 nm attributed to π → π* transition of DPPO (Fig. 5b and Table 1). **4DBSODPO** showed the least absorption peaks, in accord with its most rigid structure. The S_1_ energy of ***m*****DBSOSPO** is estimated as ~3.5 eV, only 0.1 eV less than that of **DBSO**. The situation of fluorescence spectra for ***m*****DBSOSPO** is similar that the emission profiles are identical with bathochromic shifts of about 7–13 nm compared to that of **DBSO**, which should be attributed to the electron-withdrawing effect of DPPO. However, the DPPO substitution hardly influences the characteristics of triplet excited state that all of the compounds exhibit the almost overlapped phosphorescence spectra, indicating their same T_1_ locations on **DBSO** cores. According to 0–0 transitions at ~415 nm, the T_1_ energy of ***m*****DBSOSPO** is estimated as ~3.0 eV, which is high enough to effectively confine excitons in EML. The identical optical properties of **DBT*****x*****PO** and ***m*****DBSOSPO** exclude the interference from the different energy transfer and exciton diffusion suppression efficacies on their device performance.

### DFT Simulation

To figure out the nature of electronic characteristics of ***m*****DBSOSPO**, their ground states and T_1_ states were optimized at the level of B3LYP/6-31g* with density function theory (DFT) simulation (Fig. 6 and Table 1). It is known that the frontier molecular orbitals (FMO) make the major contributions to charge gain or loss, which reflect the charge affinity and migration possibility.

The HOMO and LUMO of ***m*****DBSOSPO** are mainly localized on their **DBSO** cores, in accord with their similar optical properties, which further verify their unipolar characteristics. It is noteworthy that **3DBSOSPO** shows the deepest LUMO at −1.959 eV, slightly lower than −1.904 eV of **2DBSOSPO**, while the tendency of their HOMO is opposite. The HOMO and LUMO of **4DBSOSPO** are the shallowest among ***m*****DBSOSPO**. This should be attributed to the minimal involvement of 4-C in these orbitals, rendering the smallest influence of 4-substituted DPPO on FMO energy levels. As the result, the HOMO-LUMO energy gap of **4DBSOSPO** is the biggest, while that of **3DBSOSPO** is the smallest, which is owing to the slight conjugation extension by P = O substituted at long axis of **DBSO** core. Therefore, **2DBSOSPO** and **3DBSOSPO** have the excellent electron injecting ability, much stronger than that of **4DBSOSPO**.

**Figure 4:**
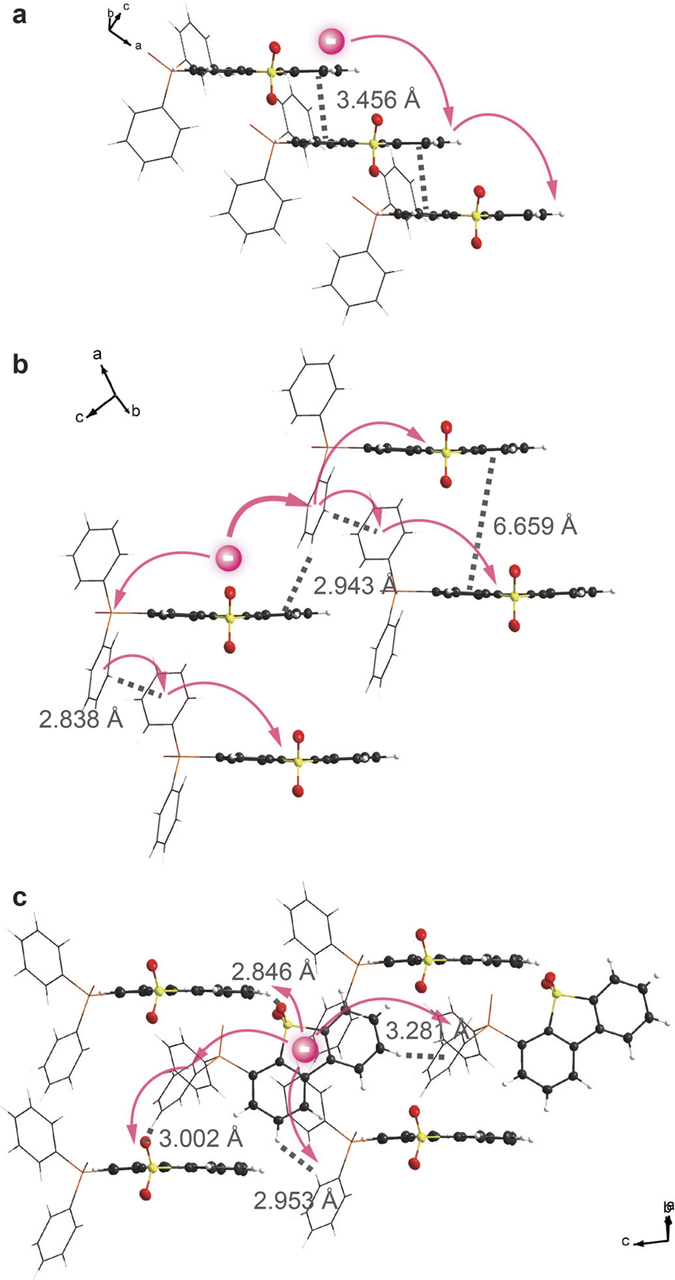
Packing diagrams of 2DBSOSPO (a) 3DBSOSPO (b) and 4DBSOSPO (c) in which DPPOs were drawn in tube mode for clarity, and their presumed intermolecular electron migration processes according to the intermolecular interactions.

**Figure 5:**
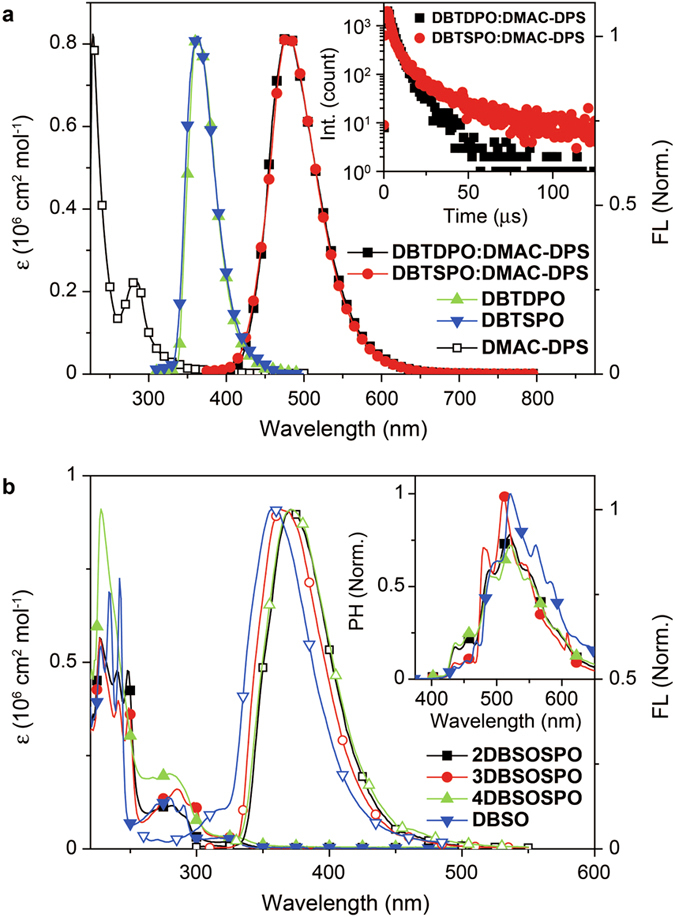
(**a**) UV absorption spectrum of **DMAC-DPS**, PL spectrum of **DBT*****x*****PO** and steady-state emission spectra and time-decay curves (inset) of **DMAC-DPS** in **DBT*****x*****PO**:**DMAC-DPS** (10%wt.) thin films; (**b**) Electronic absorption and fluorescence (FL) spectra of ***m*****DBSOSPO** and **DBSO** in CH_2_Cl_2_ (10^−6^ mol L^−1^) and low-temperature phosphorescence (PH) spectra (inset) by time-resolved technology after a delay of 300 *μ*s.

**Figure 6:**
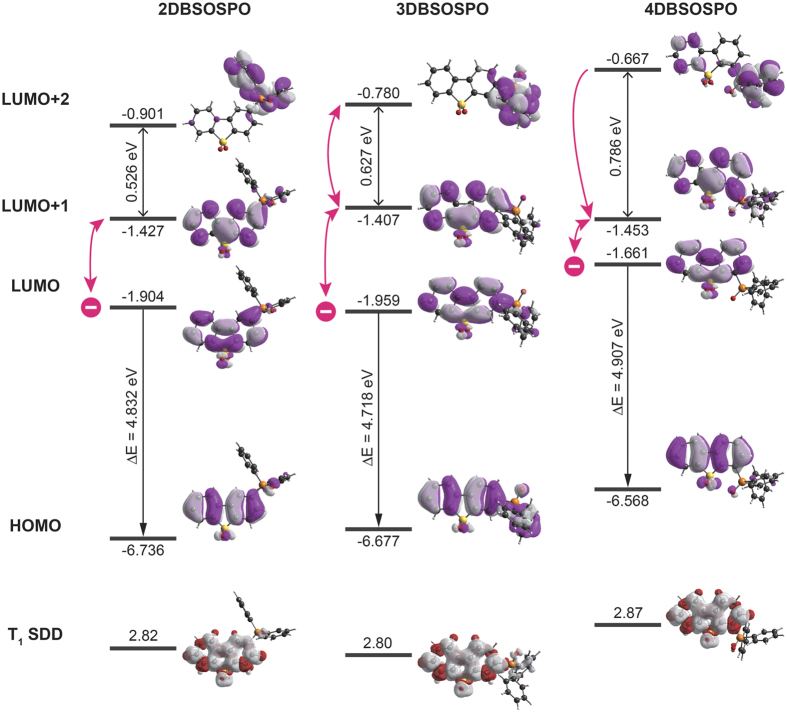
Contours and energy levels of frontier molecular orbitals and spin density distributions of triplet states for ***m*****DBSOSPO**.

The electron cloud distributions of the LUMO and LUMO + 1 orbitals for ***m*****DBSOSPO** are thoroughly localized on **DBSO** core, except for **3DBSOSPO**, whose LUMO partially extends to its DPPO; while their DPPO groups make the major contributions to their LUMO + 2 orbitals. Therefore, the predominant electron capture sites for ***m*****DBSOSPO** are **DBSO** cores, accompanied with DPPOs as transitional peripheral groups. In this sense, according to the single-crystal packing diagram of **2DBSODPO** (Fig. 4), with the effective intermolecular **DBSO**-**DBSO** interaction formed continuous electron hopping channel, its electron transporting ability should be the strongest among ***m*****DBSOSPO**. Contrarily, intermolecular electron hopping in **3DBSOSPO** and **4DBSOSPO** depends on their DPPOs as bridge groups. Owing to the involvement of DPPO in the LUMO (Fig. 4), the bidirectional electron migration between DPPO and **DBSO** in **3DBSOSPO** should be facile, also supporting a continuous energy hopping channel. However, for **4DBSOSPO**, the huge energy gap of 0.786 eV between LUMO + 1 and LUMO + 2 and the exclusion of DPPO in its LUMO render the unidirectional electron migration from DPPO to **DBSO**. It is rational that with the continuous electron hopping processes, **2DBSOSPO** and **3DBSOSPO** should be superior to **4DBSOSPO** in electron transportation.

Spin density distribution (SDD) of T_1_ states of ***m*****DBSOSPO** shows their identical T_1_ locations on **DBSO** cores, which afford their similar T_1_ value of ~2.8 eV, in accord with optical analysis data (Table 1). The similar optical properties, e.g. the HOMO-LUMO energy gaps and T_1_ energy, of ***m*****DBSOSPO** should be ascribed to the same **DBSO**-localized FMO and T_1_ states. Simultaneously, substitution position of DPPO remarkably influences the FMO energy levels and extension, which render the different electrical properties of ***m*****DBSOSPO**.

### Electrical Performance

**Figure 7:**
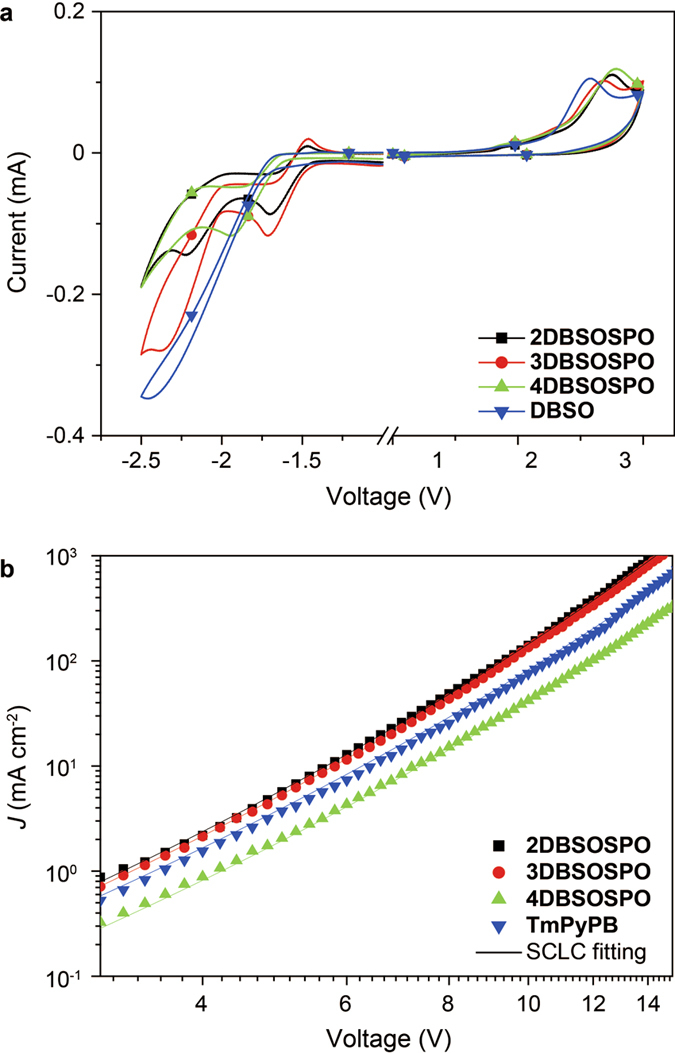
(**a**) Cyclic voltammograms of ***m*****DBSOSPO** and **DBSO** measured in CH_2_Cl_2_ at room temperature with tetrabutylammonium hexafluorophosphate (0.1 M) as electrolyte under the scanning rate of 100 mV s^−1^; (**b**) volt-ampere characteristics of ***m*****DBSOSPO** and **TmPyPB** based electron-only devices.

The electron affinity and hole blocking ability of ***m*****DBSOSPO** were evaluated by their electrochemical redox behaviors, according to the cyclic voltammogram (Fig. 7a). Compared to **DBSO**, the irreversible **DBSO**-originated anodic peaks of ***m*****DBSOSPO** shift to higher potentials with the onset voltages as high as ~2.3 V, corresponding to the extremely deep LUMO at −7.0 eV, which were even 0.3 eV lower than that of conventional hole-blocking material 2,9-Dimethyl–4,7-diphenyl-1,10-phenanthroline (**BCP**) (Table 1). In contrast to **DBSO** with single irreversible reduction peak, ***m*****DBSOSPO** showed two cathodic peaks, corresponding to the reduction of **DBSO** core and DPPO, respectively. Estimated with the onset voltages, the LUMO level of **4DBSOSPO** is −3.14 eV, only 0.06 eV lower than that of **DBSO**; while, the LUMO levels of **2DBSOSPO** and **3DBSOSPO** are dramatically reduced to about −3.3 eV, revealing their excellent electron injecting ability. It is noteworthy that contrary to the irreversible peaks of **4DBSOSPO**, owing to the electron-withdrawing inductive effect of DPPO at 2 and 3 positions, the **DBSO**-attributed peaks of **2DBSOSPO** and **3DBSOSPO** become reversible, indicating their stable and cyclical electron capture ability.

As ETMs, the electron mobility of ***mDBSOSPO***is one of the most important indicators. The single-layer nominal electron-only devices of ***m*****DBSOSPO** were fabricated with configuration of ITO|LiF (1 nm)|***m*****DBSOSPO** (100 nm)|LiF (1 nm)|Al to estimate their intrinsic electron transporting ability, where LiF served as electron-injecting layers. Volt-ampere characteristics of these electron-only devices are shown in Fig. 7b. The current density (*J*) of **4DBSOSPO**-based devices was the lowest at the same voltages among these devices; while, **2DBSOSPO** and **3DBSOSPO** endowed their devices with the similar *I-V* curves. The stronger electron transporting ability of **2DBSOSPO** and **3DBSOSPO** can be owing to their higher electron affinity and the continuous intermolecular electron hopping channels, as demonstrated by DFT simulation and single-crystal results. Significantly, according to the model of field-dependent space charge limited current (FD-SCLC), electron mobility (*μ*_e_) of ***m*****DBSOSPO** were estimated as high as 3.96 × 10^−3^, 3.69 × 10^−3^ and 1.45 × 10^−3^ cm^2^ V^−1^ s^−1^, respectively (Table 1). The mobility of **2DBSOSPO** and **3DBSOSPO** was even higher than that of the most popular ETM 1,3,5-tri[(3-pyridyl)-phen-3-yl]benzene (**TmPyPB**) with high triplet energy. making them outstanding among high-energy-gap ETMs reported so far.

Therefore, in accord with DFT simulation results and intermolecular interactions, **2DBSOSPO** and **3DBSOSPO** show their superiority in electron injection and transportation. The correlation of the substitution position of DPPO and its inductive effect and intermolecular interactions can be utilized to feasibly modulate electrical properties at the same time of configuration adjustment. With the LUMO lower than −3.0 eV and the electron mobility by the level of 10^−3^ cm^2^ V^−1^ s^−1^, the charge flux balance in EML of ***m*****DBSOSPO**-based device can be expected.

### EL performance of blue TADF devices

**Figure 8:**
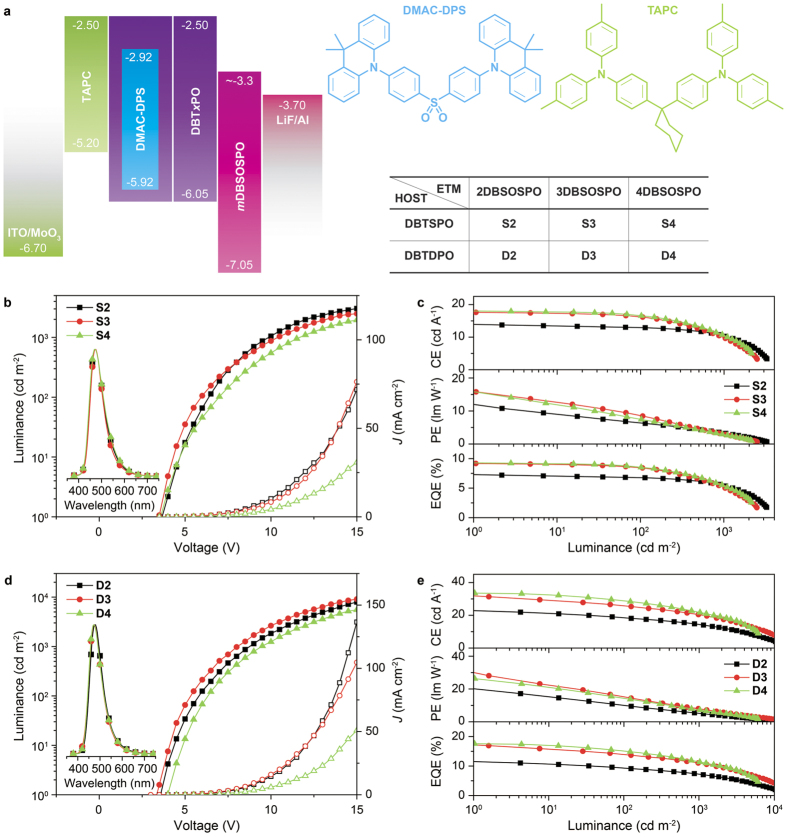
(**a**) Device structure and energy level diagram of **DMAC-DPS** based blue TADF OLEDs; (**b,d**) Luminance-current density (*J*)-voltage characteristics and (c and e) efficiencies-luminance curves of ***m*****DBSOSPO**-based blue TADF devices (**b,c**) for **DBTSPO**-based devices **S2**–**S4**; while (**d,e**) for **DBTDPO**-based devices **D2**–**D4**).

To figure out the influence of intralayer and interlayer compatibility on EL performance of blue TADF diodes, two series of devices were fabricated with a four-layer configuration of ITO|MoO_3_ (8 nm)|TAPC (70 nm)|**DBT*****x*****PO**:**DMAC-DPS** (10%, 20 nm)|**DBT*****x*****PO** (5 nm)|***m*****DBSOPO** (35 nm)|LiF (1 nm)|Al, in which TAPC was di-[4-(N,N-ditolyl-amino)-phenyl]cyclohexane as hole transporting layer. A neat **DBT*****x*****PO** layer was inserted between EML and ETL to alleviate interfacial effects, whose thickness was optimized as 5 nm in order to confine exciton in EML. Therefore, when **DBTSPO** served as host, the devices **S2**, **S3** and **S4**, collectively named **Sn**, used **2DBSOSPO**, **3DBSOSPO** and **4DBSOSPO** as electron transporting layer, respectively; while, devices **D2**, **D3** and **D4**, collectively named **Dn**, employed **DBTDPO** as the host (Fig. 8a). The vertical comparison between two series of devices with different hosts reflected the influence of intralayer compatibility of EML on EL performance. Meanwhile, on account of the identical optical properties of ***m*****DBSOPO**, horizontal comparative research on the differences in EL performance of each device series should be attributed to the discrepancy of ***m*****DBSOPO** in electrical characteristics and compatibility with host. All of the devices showed the pure emissions from **DMAC-DPS** with peaks at 472 nm, revealing the effective exciton confinement on the dopant by the high T_1_ energy of **DBT*****x*****PO** and ***m*****DBSOSPO** (insets in Figs 7d and 8b).

The driving voltages of **S3** was the lowest among **Sn** as 3.5 V for onset and 6.0 V at 100 cd m^−2^ (Fig. 8b and Table S1), which were 0.5 V lower than those of **S2**. However, when driving voltage exceeded 8 V, the luminance of **S2** was larger than that of **S3**. **S4** revealed the highest driving voltages, which were 0.5–1.5 V higher than those of **S3**. The tendency of driving voltages for **Dn** was similar that **D3** showed the lowest driving voltages of 3.5 V for onset, 5.5 and 8.5 V at 100 and 1000 cd m^−2^, which were 0.5 V lower than those of **D2** and 1 V lower than those of **D4** (Fig. 8d and Table S1). Simultaneously, *J* of **Sn** was in the order of **S2** ≈ **S3** > **S4**, which was similar to the situation of **Dn**. Therefore, the driving voltage and charge flux in the devices were coincident with the electron affinity and *μ*_e_ of the corresponding ETM ***m*****DBSOSPO**, which implies the majority carrier of hole in these devices and recombination zone adjacent the interface between EML and ETL. In this case, the suppression of interfacial quenching would be crucial to high efficiencies. In this case, it is noticeable that when *J* more than 8.5 mA cm^−2^, the luminance of **S3** turned to less than that of **S2**, indicating the more serious quenching for the combination of **DBTSPO** and **3DBSOSPO**.

The efficiencies directly showed the effect of interlayer and intralayer compatibility on the device performance (Figs. 7d and 8c and Table S1). The maximum efficiencies of **S3** and **S4** were comparable as 17.6 and 17.9 cd A^−1^ for CE, 15.8 and 14.1 lm W^−1^ for power efficiency (PE) and 9.2% for EQE, which were much higher than those of **S2** as 13.7 cd A^−1^, 10.8 lm W^−1^ and 7.2%. The lowest efficiencies of **S2** should be attributed to the worst compatibility between **2DBSOSPO** and **DBTSPO** regarding to their opposite configurations and much different polarity. It is also noteworthy that in spite of its worst electrical performance, **4DBSOSPO** still endowed its devices with the high efficiencies, in virtue of its strongest compatibility with *ortho*-linked **DBT*****x*****PO**. Nevertheless, the efficiency roll-offs of **S2** were the lowest as 6 and 25% for CE, 43 and 70% for PE and 6 and 25% for EQE at 100 and 1000 cd m^−2^, respectively, which should be owing to its most balanced charge flux supported by **2DBSOSPO**. The situation for **Dn** was similar that **D3** and **D4** revealed the similar maximum efficiencies of 31.3 and 33.5 cd A^−1^, 28.1 and 26.3 lm W^−1^ and 16.9 and 17.4%, respectively, which were about 1.5 folds of those of **D2**. Differently, the efficiency roll-offs of **Dn** were almost the same as 15 and 35% for CE, 45 and 70% for PE and 15 and 35% for EQE at 100 and 1000 cd m^−2^, respectively, which was originated from their similar interfacial quenching effects due to the incompatibility between **DBTDPO** and ***m*****DBSOSPO**. Simultaneously, at 100 cd m^−2^, **DBTSPO** supported its devices **Sn** with the efficiency roll-offs only a half of those of **Dn**, which was obviously owing to the suppressed interfacial quenching by favorable compatibility between **DBTSPO** and ***m*****DBSODPO**. Nevertheless, compared with **Sn**, **DBTDPO** endowed **Dn** with the maximum efficiencies dramatically improved for 50%, correspondingly, which should be ascribed to the stronger compatibility between **DBTDPO** and **DMAC-DPS**. Then, due to their limited luminance at high *J*, the efficiency roll-offs of **S3** and **S4** at 1000 cd m^−2^ became higher than those of **Dn**. The combined utilization of **DBTDPO** as host and **3DBSOPO** or **4DBSOSPO** as ETM endowed their devices with the impressive EL performance, e.g. the maximum EQE beyond 15%, among the best blue TADF devices reported so far.

In general, the higher efficiencies of **Dn** than those of **Sn** manifested that the intralayer compatibility between host and dopant is the primary issue should be considered. Meanwhile, the interlayer compatibility between EML and ETL would have remarkable influence on the efficiency stability, as indicated by the lower efficiency roll-offs at 100 cd m^−2^ of **Sn** than those of **Dn**.

## Conclusions

A series of dibenzothiophene-based hosts and ETMs **DBT*****x*****PO** and ***m*****DBSOSPO** were utilized to realize high-performance blue TADF devices with optimized intralayer and interlayer compatibility. AFM images showed the more uniform dispersion of **DMAC-DPS** in **DBTDPO**, revealing their stronger compatibility, which further endows **DBTDPO**:**DMAC-DPS** films with the higher PLQY and reduced emission lifetime. The optical properties of ***m*****DBSOSPO** are well-controlled with similar S_1_ and T_1_ for effective exciton confinement in EML, while, their electrical properties are enhanced by DPPO substitution position, rendering the LUMO levels lower than –3.0 eV for effective electron injection and high *μ*_e_ by the order of 10^−3^ cm^2^ V^−1^ s^−1^. Through employing **DBTDPO** as host and **3DBSOPO** or **4DBSOSPO** as ETM, the impressive EL performance, e.g. the maximum EQE beyond 17%, were realized, which was among the best results for blue TADF devices. On the basis of comprehensive comparison on the influence of host and ETM variation on the EL performances, it is showed that intralayer compatibility between host and dopant is the primary determinant of device efficiencies due to its correlation with EML-inside quenching. Simultaneously, the interlayer compatibility between EML and ETL would influence the interfacial quenching effects, thereby having remarkable effects on efficiency roll-off reduction. This work verified the significance of host-dopant compatibility and interfacial optimization on developing high-performance blue TADF diodes.

## Methods

### Materials and Instruments

All the reagents and solvents used for the synthesis of the compounds were purchased from Aldrich and Acros companies and used without further purification.

^1^H NMR spectra were recorded using a Varian Mercury plus 400 NB spectrometer relative to tetramethylsilane (TMS) as internal standard. Molecular masses were determined by a FINNIGAN LCQ Electro-Spraying Ionization-Mass Spectrometry (ESI-MS), or a MALDI-TOF-MS. Elemental analyses were performed on a Vario EL III elemental analyzer. The crystal suitable for single-crystal XRD analysis was obtained through slowly diffusing hexane into dichloromethane solution of *m*DBSOSPO at room temperature. All diffraction data were collected at 295 K on a Rigaku Xcalibur E diffractometer with graphite monochromatized Mo Kα (λ = 0.71073 Å) radiation in ω scan mode. All structures were solved by direct method and difference Fourier syntheses. Non-hydrogen atoms were refined by full-matrix least-squares techniques on F2 with anisotropic thermal parameters. The hydrogen atoms attached to carbons were placed in calculated positions with C−H = 0.93 Å and U(H) = 1.2 Ueq(C) in the riding model approximation. All calculations were carried out with the SHELXL97 program. Absorption and photoluminescence (PL) emission spectra of the target compound were measured using a SHIMADZU UV-3150 spectrophotometer and a SHIMADZU RF-5301PC spectrophotometer, respectively. Thermogravimetric analysis (TGA) and differential scanning calorimetry (DSC) were performed on Shimadzu DSC-60A and DTG-60A thermal analyzers under nitrogen atmosphere at a heating rate of 10 °C min^−1^. Cyclic voltammetric (CV) studies were conducted using an Eco Chemie B. V. AUTOLAB potentiostat in a typical three-electrode cell with a platinum sheet working electrode, a platinum wire counter electrode and a silver/silver chloride (Ag/AgCl) reference electrode. Tetrabutylammonium hexafluorophosphate was used as electrolyte with a concentration of 0.1 mol L^−1^. All electrochemical experiments were carried out under a nitrogen atmosphere at room temperature in dichloromethane. Phosphorescence spectra were measured in dichloromethane using an Edinburgh FPLS 920 fluorescence spectrophotometer at 77 K cooling by liquid nitrogen with a delay of 300 *μ*s using Time-Correlated Single Photon Counting (TCSPC) method with a microsecond pulsed Xenon light source for 10 *μ*s-10 s lifetime measurement involving scatterer for correction, the synchronization photomultiplier for signal collection and the Multi-Channel Scaling Mode of the PCS900 fast counter PC plug-in card for data processing.

### General procedure of Pd-catalyzed P-C coupling

In Ar_2_, 2.95 g (10 mmol) of *m*DBSOBr, 1.23 g of NaOAc (15 mmol), 0.0112 g of Pd(OAc)_2_ (0.05 mmol) and 2.63 mL of Ph_2_PH (15 mmol) were dissolved in 25 mL of DMF. The mixture was stirred for 24 h at 130 °C. The reaction was then quenched by adding 25 mL of water, and extracted with 3 × 25 mL of CH_2_Cl_2_. The organic layer was combined, concentrated to 10 mL and cooled to 0 °C. Then, 4 ml of 30% H_2_O_2_ was added to the mixture and stirred for 8 h. The system was extracted with 3 × 10 mL of CH_2_Cl_2_. The organic layer was dried with anhydrous sodium sulfate. The solvent was removed in *vacuo*. The residue was purified by flash column chromatography to the title compounds.

*2-(diphenylphosphoryl)dibenzothiophene sulfone* (**2DBSOSPO**): white powder with a yield of 40%. ^1^H NMR (TMS, CDCl_3_, 400 M Hz): *δ* = 8.392 (d, *J* = 11.6 Hz, 1H), 7.854 (q, *J*_1_ = 9.0, *J*_2_ = 17.0 Hz, 3H), 7.683 (q, *J*_1_ = 7.8, *J*_2_ = 13.0 Hz, 5 H), 7.602 (m, 4 H), 7.544 ppm (t, *J* = 7.4 Hz, 4 H); LDI-TOF: m/z (%): 416 (100) [M^+^]; elemental analysis (%) for C_24_H_17_O_3_PS: C 69.22, H 4.11, O 11.53, S 7.70; found: C 69.25, H 4.09, O 11.65, S 7.77.

*3-(diphenylphosphoryl)dibenzothiophene sulfone* (**3DBSOSPO**): white powder with a yield of 35%. ^1^H NMR (TMS, CDCl_3_, 400 M Hz): *δ* = 8.148 (t, *J* = 9.6 Hz, 1H), 7.941 (t, *J* = 5.6 Hz, 2H), 7.844 (t, *J* = 8.4 Hz, 2H), 7.680 (q, *J*_1_ = 7.0, *J*_2_ = 12.2 Hz, 5H), 7.600 (m, 3H), 7.513 (t, *J* = 7.4 Hz, 4H); LDI-TOF: m/z (%): 416 (100) [M^+^]; elemental analysis (%) for C_24_H_17_O_3_PS: C 69.22, H 4.11, O 11.53, S 7.70; found: C 69.23, H 4.11, O 11.70, S 7.81.

*4-(diphenylphosphoryl)dibenzothiophene sulfone* (**4DBSOSPO**): white powder with a yield of 35%. ^1^H NMR (TMS, CDCl_3_, 400 M Hz): *δ* = 7.997 (d, *J* = 2.8 Hz, 1H), 7.795 (q, *J*_1_ = 7.6, *J*_2_ = 12.0 Hz, 5H), 7.693 (q, *J*_1_ = 8.4, *J*_2_ = 18.0 Hz, 3H), 7.587 (t, *J* = 6.8 Hz, 3H), 7.493 (t, *J* = 7.4 Hz, 5H); LDI-TOF: m/z (%): 416 (100) [M^+^]; elemental analysis (%) for C_24_H_17_O_3_PS: C 69.22, H 4.11, O 11.53, S 7.70; found: C 69.25, H 4.13, O 11.68, S 7.76.

### DFT Calculations

DFT computations were carried out with different parameters for structure optimizations and vibration analyses. The ground states and triplet states of molecules in vacuum were optimized with assistance of single crystal structures by the restricted and unrestricted formalism of Beck’s three-parameter hybrid exchange functional^58^ and Lee, and Yang and Parr correlation functional^59^ (B3LYP)/ 6–31G(d) respectively. The fully optimized stationary points were further characterized by harmonic vibrational frequency analysis to ensure that real local minima had been found without imaginary vibrational frequency. The total energies were also corrected by zero-point energy both for the ground state and triplet state. The Spin Density Distributions were visualized with Gaussview 3.0. All computations were performed using the Gaussian 03 package.

### Device Fabrication and Testing

Before loading into a deposition chamber, the ITO substrate was cleaned with detergents and deionized water, dried in an oven at 120 °C for 4 h, and treated with oxygen plasma for 3 min. Devices were fabricated by evaporating organic layers at a rate of 0.1–0.2 nm s^−1^ onto the ITO substrate sequentially at a pressure below 4 × 10^−4^ Pa. Onto the electron-transporting layer, a layer of LiF with 1 nm thickness was deposited at a rate of 0.1 nm s^−1^ to improve electron injection. Finally, a 100-nm-thick layer of Al was deposited at a rate of 0.6 nm s^−1^ as the cathode. The emission area of the devices was 0.09 cm^2^ as determined by the overlap area of the anode and the cathode. After fabrication, the devices were immediately transferred to a glove box for encapsulation with glass cover slips using epoxy glue. The EL spectra and CIE coordinates were measured using a PR655 spectra colorimeter. The current-density-voltage and brightness–voltage curves of the devices were measured using a Keithley 4200 source meter and a calibrated silicon photodiode. All the measurements were carried out at room temperature under ambient conditions. For each structure, five devices were fabricated to confirm the performance repeatability. To make conclusions reliable, the data reported herein were most close to the average results.

## Additional Information

**How to cite this article**: Duan, C. *et al*. Optimizing the Intralayer and Interlayer Compatibility for High-Efficiency Blue Thermally Activated Delayed Fluorescence Diodes. *Sci. Rep*. **6**, 19904; doi: 10.1038/srep19904 (2016).

## Supplementary Material

Supplementary Information

## Figures and Tables

**Table 1:** Physical properties of DBSO derivatives.

Compound	Absorption (nm)	Emission (nm)	S_1_ (eV)	T_1_ (eV)	*T*_g_/*T*_m_/*T*_d_ (^o^C)	HOMO (eV)	LUMO (eV)	RE^g^ (eV)	*μ*_e_^h^ (cm^2^/V/s)
**DBSO**	324, 291, 278, 269, 242, 234, 228^a^, 337, 283, 205^b^	358^a^ 402^b^	3.60^c^ 4.85^d^	3.00^e^ 2.86^d^	−/−/207	−6.56^f^ −6.67^d^	−3.08^f^ −1.82^d^	0.4261	−
**2DBSODPO**	330, 282, 249, 240, 227^a^, 335, 294, 285, 275, 254^b^	371^a^ 381^b^	3.51^c^ 4.77^d^	2.98^e^ 2.82^d^	151/221/367	−7.10^f^ −6.76^d^	−3.29^f^ −1.99^d^	0.5505	3.96 × 10^−3^
**3DBSODPO**	325, 298, 285, 249, 241, 228^a^, 334, 303, 290, 277, 255, 246^b^	365^a^ 378^b^	3.53^c^ 4.71^d^	2.99^e^ 2.80^d^	−/−211/373	−7.05^f^ −6.74^d^	−3.31^f^ −2.03^d^	0.5075	3.69×10^-3^
**4DBSODPO**	332, 282, 228^a^ 335, 296, 282, 253^b^	371^a^ 375^b^	3.51^c^ 4.85^d^	2.97^e^ 2.87^d^	−/−262/375	−7.09^f^ −6.63^d^	−3.14^f^ −1.78^d^	0.5291	1.45 × 10^−3^

^a^In CH2Cl2 (10–6 mol L-1);

^b^in film;

^c^estimated according to the absorption edges;

^d^DFT calculated results;

^e^calculated according to the 0–0 transitions of the phosphorescence spectra;

^f^calculated according to the equation HOMO/LUMO = 4.78 + onset voltage^60^;

^g^reorganization energy of electron;

^h^electron mobility estimated by *I-V* characteristics of electrononlydevices according filed-dependent SCLC model^61^.
