# Supplementary material for: Optimizing the Intralayer and Interlayer Compatibility for High-Efficiency Blue Thermally Activated Delayed Fluorescence Diodes
Source: Sci Rep. 2016 Jan 29;6:19904. doi: 10.1038/srep19904 (PMC4731786; doi:10.1038/srep19904)
Supplement: Supplementary Information [file srep19904-s1.doc]

**Supplementary Information**

### Optimizing the Intralayer and Interlayer Compatibility for High-Efficiency Blue Thermally Activated Delayed Fluorescence Diodes

Chunbo Duan,^a^ Chaochao Fan,^a^ Ying Wei,^a,b^ Fuquan Han,^a^ Wei Huang*^b^ and Hui Xu,*^a,b^

^a^ Key Laboratory of Functional Inorganic Material Chemistry, Ministry of Education, Heilongjiang University, 74 Xuefu Road, Harbin 150080 (P. R. China)

^b^ Key Laboratory of Flexible Electronics (KLOFE) & Institute of Advanced Materials (IAM), Jiangsu National Synergetic Innovation Center for Advanced Materials (SICAM), Nanjing Tech University (NanjingTech), 30 South Puzhu Road, Nanjing 211816 (P.R. China)


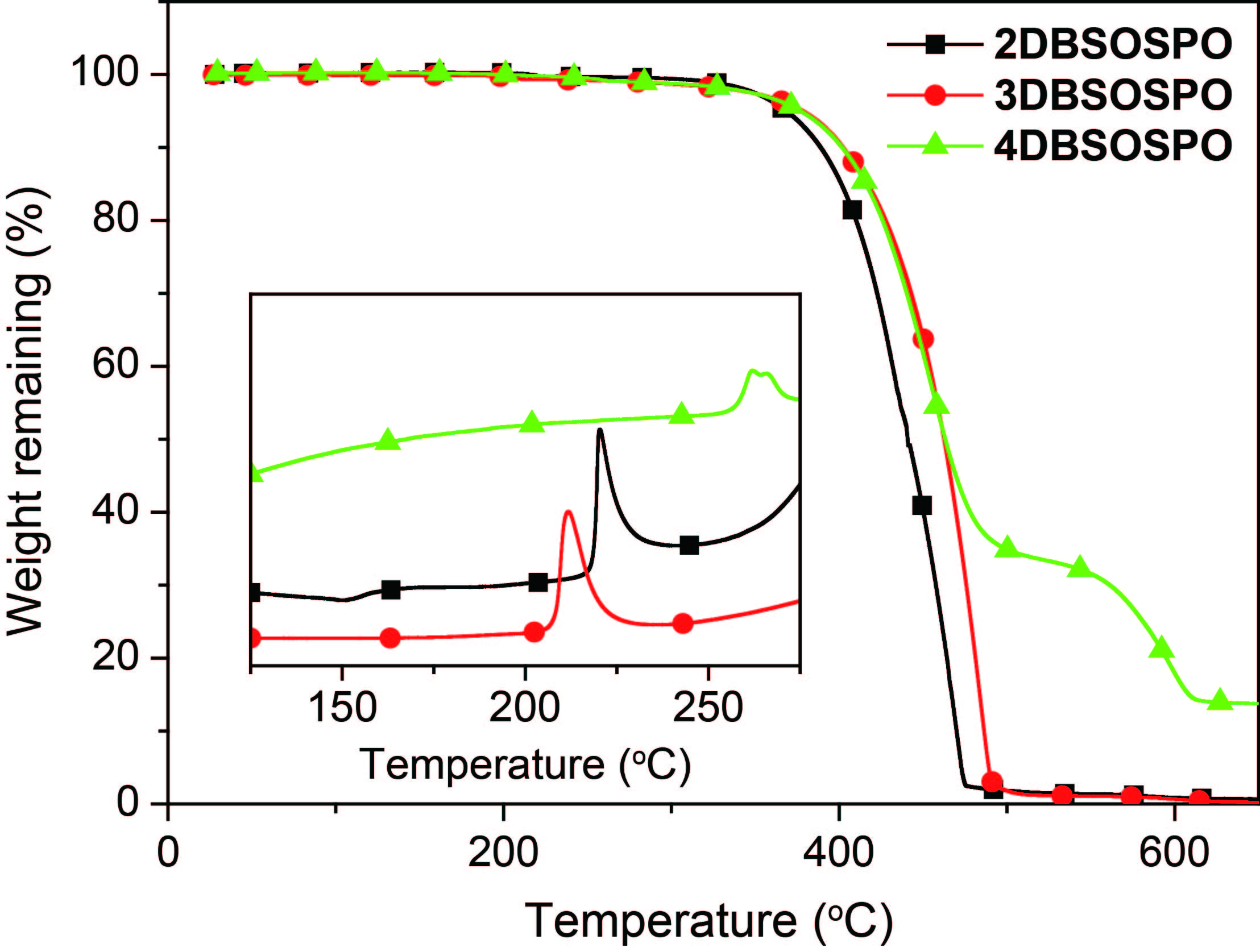


**Figure S1.** TGA and DSC (inset) curves of ***m*DBSOSPO**.

### Table S1. EL performance of DBT*x*PO and *m*DBSOSPO based blue TADF diodes.

| Device | Operating Voltage^a^ (V) | Maximum Efficiencies^b^ | Efficiency Roll-Offs^c^ (%) | | |
| --- | --- | --- | --- | --- | --- |
|  |  |  | CE | PE | EQE |
| **S2** | <4.0, <6.5, <10.0 | 13.7, 10.8, 7.2 | 6, 25 | 43, 70 | 6, 25 |
| **S3** | <3.5, <6.0, <10.5 | 17.6, 15.8, 9.2 | 8, 45 | 46, 82 | 9, 46 |
| **S4** | <4.0, <7.0, <12.0 | 17.9, 14.1, 9.2 | 7, 44 | 48, 82 | 8, 44 |
| **D2** | <4.0, <6.0, <9.0 | 21.9, 17.2, 11.0 | 17, 36 | 45, 72 | 17, 35 |
| **D3** | <3.5, <5.5, <8.5 | 31.3, 28.1, 16.9 | 19, 37 | 48, 74 | 19, 37 |
| **D4** | <4.0, <6.5, <9.5 | 33.5, 26.3, 17.4 | 15, 35 | 48, 73 | 14, 34 |

a) In the order of onset, 100 and 1000 cd m^-2^; b) in the order of C.E. (cd A^-1^), P.E. (lm W^-1^) and E.Q.E. (%); c) in the order of 100 and 1000 cd m^-2^.
